# A Cilia Independent Role of Ift88/Polaris during Cell Migration

**DOI:** 10.1371/journal.pone.0140378

**Published:** 2015-10-14

**Authors:** Christopher Boehlke, Heike Janusch, Christoph Hamann, Christian Powelske, Miriam Mergen, Henriette Herbst, Fruzsina Kotsis, Roland Nitschke, E. Wolfgang Kuehn

**Affiliations:** 1 Department of Nephrology, University Hospital, Freiburg, Germany; 2 Life Imaging Center, Center for Biosystems Analysis, Albert-Ludwig-University, Freiburg, Germany; 3 Center for Biological Signaling Studies (bioss), Albert-Ludwig-University, Freiburg, Germany; Justus-Liebig-University Giessen, GERMANY

## Abstract

Ift88 is a central component of the intraflagellar transport (Ift) complex B, essential for the building of cilia and flagella from single cell organisms to mammals. Loss of Ift88 results in the absence of cilia and causes left-right asymmetry defects, disordered Hedgehog signaling, and polycystic kidney disease, all of which are explained by aberrant ciliary function. In addition, a number of extraciliary functions of Ift88 have been described that affect the cell-cycle, mitosis, and targeting of the T-cell receptor to the immunological synapse. Similarly, another essential ciliary molecule, the kinesin-2 subunit Kif3a, which transports Ift-B in the cilium, affects microtubule (MT) dynamics at the leading edge of migrating cells independently of cilia. We now show that loss of Ift88 impairs cell migration irrespective of cilia. Ift88 is required for the polarization of migrating MDCK cells, and Ift88 depleted cells have fewer MTs at the leading edge. Neither MT dynamics nor MT nucleation are dependent on Ift88. Our findings dissociate the function of Ift88 from Kif3a outside the cilium and suggest a novel extraciliary function for Ift88. Future studies need to address what unifying mechanism underlies the different extraciliary functions of Ift88.

## Introduction

Ift88 is an essential protein within cilia that has played a pivotal role in unveiling the function of cilia in mammalian development and disease [1]. The Oak Ridge Polycystic Kidney (ORPK) mouse was described in a mutagenesis screen and characterized by scruffy fur, skeletal abnormalities and polycystic kidneys [2,3]. The mutated gene *Tg737* was subsequently recognized as an orthologue to *OSM5*, a ciliary protein in *Caenorhabditis elegans [4]*, and to *ift88*, a flagellar component in *Chlamydomonas reinhardtii [5]*. Intraflagellar transport (Ift) proteins assemble into macromolecular complexes that travel along the flagellar axoneme, transported by kinesin or dynein motors [6,7]. Ift88 is part of Ift complex B which is moved by kinesin in the anterograde direction along flagella and is universally required for the formation of this organelle.

The *Tg737* mutation in the ORPK mouse is a hypomorphic allele. Targeted disruption of this gene results in embryonic lethality between E10.5 and 11.5 [8]. The Ift88 deficient embryos display several features of disturbed cilia function: defects in left- right asymmetry are a consequence of a deficiency to generate a left sided flow at the embryonic node. Skeletal defects are attributed to deficient Sonic Hedgehog (SHH) signaling, a signal transduction pathway that in vertebrates crucially relies on trafficking of its core components within cilia [9]. In addition, cilia independent roles have been described for Ift88. They include a role in mitosis, a stage of the cell cycle when cilia are not present: work in fibroblasts from ORPK mice and in zebrafish demonstrates that Ift88 is required for the formation of astral MTs and the lack of Ift88 results in misalignment of the mitotic spindle [10]. Ift88 has additional functions during cell division: Ift88 influences cell-cycle progression during G1-S transition [11]. A cell-cycle independent function was described in lymphocytes, where cilia are never formed. Lymphocyte Ift88 is part of the endocytotic recycling machinery, which targets the T-cell receptor to the immunological synapse [12].

In light of the complex phenotype in embryos lacking Ift88, it is possible that not all of the observed alterations are related to cilia generated flow, ciliary Hedgehog signalling or the cell-cycle. One possibility is that cell migration is disturbed during embryogenesis of Ift88 mutant animals. Previously, it has been reported that fibroblasts from hypomorphic ORPK (*Ift88* ^*Tg737Rpw*^) mice [13], which grow stumpy cilia, display disordered migratory behavior in response to PDGF-AA, a chemotactic agent known to signal through cilia [14,15,16]. Indeed, wound healing experiments in the skin of ORPK mice revealed delayed wound closure. However, since ORPK cells have cilia, albeit of dysmorphic structure, these observations do not clarify if Ift88 has a non-ciliary function in cell migration.

MTs are affected by Ift88 during mitosis [10]. In addition MTs have a fundamental role in the establishment of cellular asymmetry in migrating cells [17,18]. MTs are primarily nucleated at the MTOC as well as at additional sites including the Golgi. In migrating cells a subset of MTs radiate into the leading edge where they deliver proteins affecting the protein composition at the leading edge, MT behavior, and the actin myosin network, as well as vesicles. Static and dynamic alterations of MTs can be observed. They include posttranslational modifications such as tyrosination, acetylation and glutamylation, as well as MT growth, pausing and shortening. Previous data show that Kif3a, a subunit of kinesin 2 which transports Ift88 in the cilium, affects MT dynamics and is thus involved in the regulation of cell migration [19]. We studied the question if Ift88 affects migration independently of its ciliary function and how the MT network is affected by Ift88. We analyzed transgenic MDCK cells expressing tetracycline inducible shRNA against Ift88 in wound healing experiments. We now report that depletion of Ift88 perturbs sheet migration in a cilia- and cell-cycle independent manner. Depletion of Ift88 interfered with the polarization of migrating single cells. Ift88 deficient cells displayed reduced numbers of MTs at the leading edge, but this was not associated with altered dynamic instability, changes in posttranslational modifications of MTs or MT nucleation at the centrosome. Together, these data demonstrate that Ift88 facilitates migration in a cilia- and cell-cycle independent manner by promoting directional polarity and altering the MT cytoskeleton.

## Results

### Ift88 is involved in cell migration in ciliated epithelial cells

**Fig 1:**
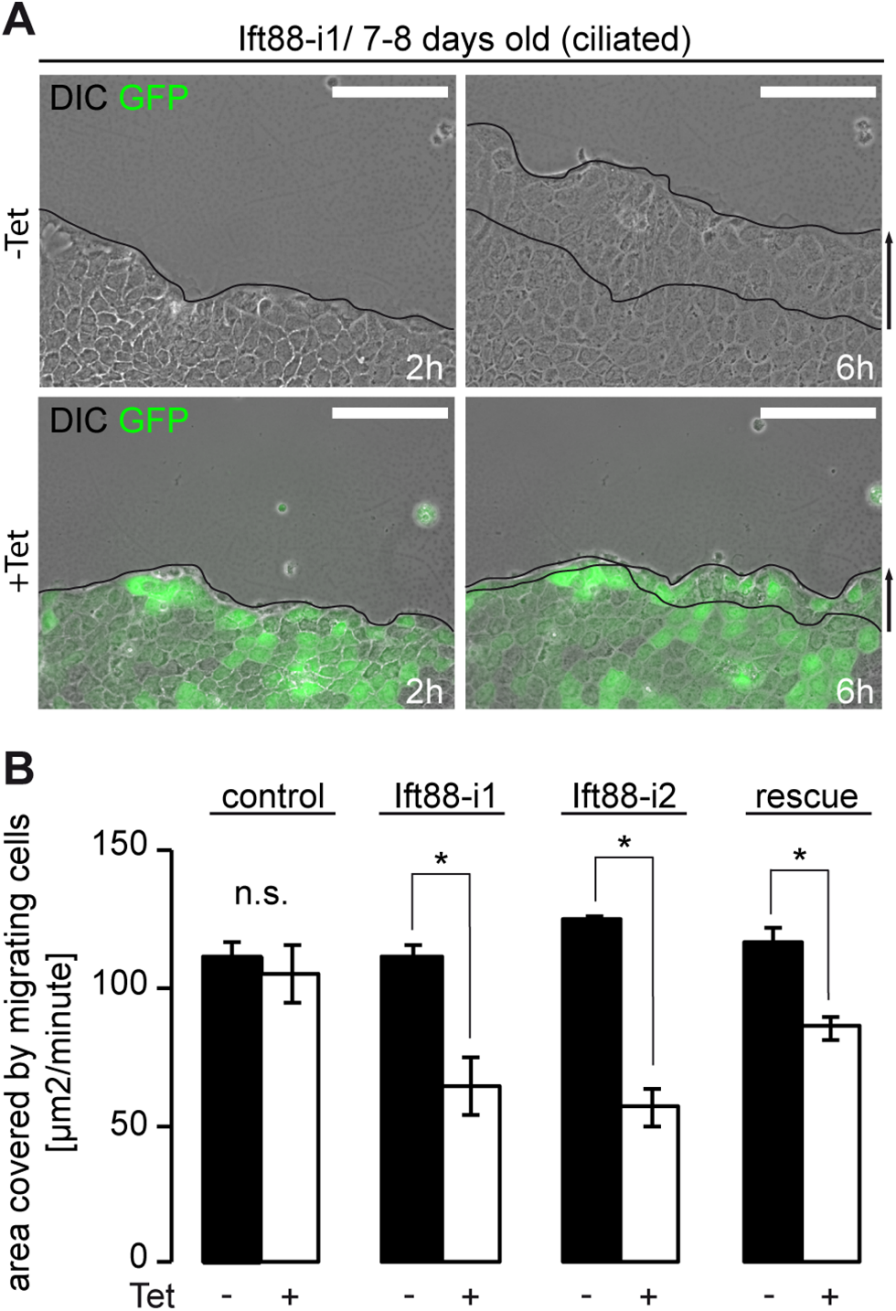
Ift88 expedites cell migration in ciliated MDCK cells. **(A)** Ift88-i1 cells were grown to confluency for 7–8 days (ciliated stage) and subjected to wounding. Cells depleted of Ift88 by inducible shRNA (+Tet) migrate slower compared with non-induced control cells (-Tet). The leading edge is shown after 2h and 6h. Scale bars: 100μm. **(B)** Quantification of migration speed in different cell lines expressing different tetracycline inducible shRNAs. No significant (n.s.) reduction in migration speed is observed in cells expressing unspecific shRNA (control; -Tet: 111.5 ±5.8 μm^2^/minute vs. 105.3 ±10.2 μm^2^/minute, p = 0.46, n = 4). Two independent cell lines expressing different shRNAs against Ift88 show significantly reduced migration speed: Ift88-i1: -Tet: 111.0 ±4.7 μm^2^/minute vs. +Tet: 64.6 ±10.3 μm^2^/minute, p<0.01, n = 4; Ift88-i2: -Tet: 124.7 ±1.2 μm^2^/minute vs. +Tet: 56.7 ±6.8 μm^2^/minute, p<0.01, n = 3. Migration speed is partially restored in Ift88-i1 cells expressing non-degradable Ift88 mRNA (Ift88-i1.rescue): -Tet: 116.5 ±5.5 μm^2^/minute vs. +Tet: 85.8 ±4.3 μm^2^/minute, p<0.01, n = 4.

To study cell migration we first conducted wound healing assays in MDCK cells 7–8 days after seeding. We took advantage of a previously established cell line expressing tetracycline inducible shRNA against Ift88 [20] and compared wound closure after scratching in Ift88 depleted conditions (+tetracycline) with controls (-tetracycline). Ift88 depletion resulted in a markedly reduced ability to close the epithelial gap (Fig 1A and S1A Fig), as quantified by measuring the area filled by migrating cells over time (Fig 1B). Control cells expressing an unspecific shRNA did not show any difference in migration area upon incubation with tetracycline. To exclude off-target effects of the shRNA we repeated the experiment with cells expressing a second shRNA against Ift88 encoding a different target sequence (Fig 1B and S1B Fig). Again, migration was perturbed after depletion of Ift88. We further conducted rescue experiments and overexpressed non-degradable Ift88 in the shRNA expressing cell line (S1C Fig). This partly reversed the migration phenotype (Fig 1B). These observations demonstrate that Ift88 facilitates the migration of ciliated epithelial cells during wound closure of a confluent epithelial layer.

### MDCK cells lose cilia during collective cell migration

**Fig 2:**
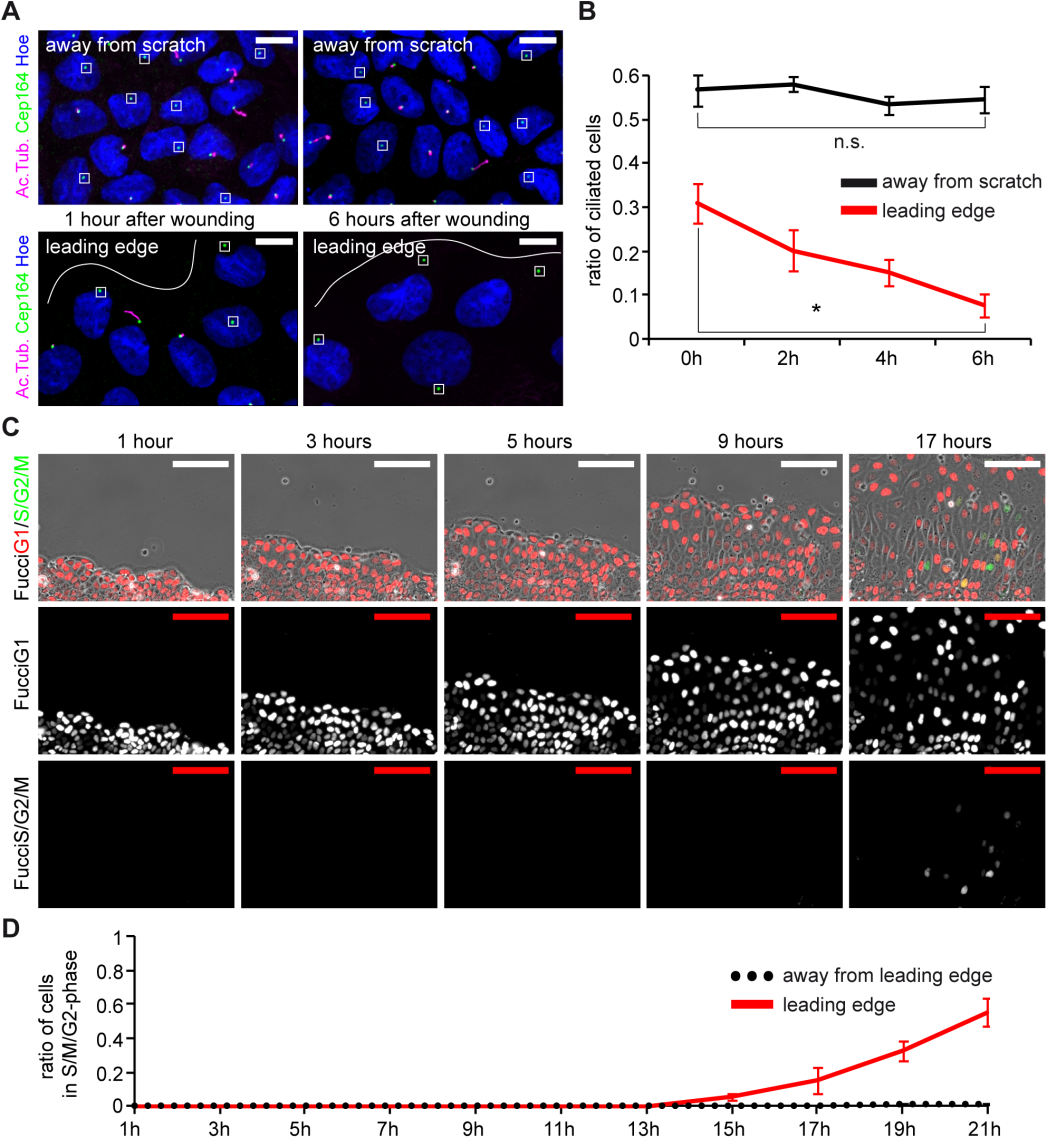
MDCK cells lose cilia during sheet migration. **(A)** Confluent layers of ciliated MDCK cells (7 days after seeding) were scratched, fixed immediately (0h) or 6h after wounding, then stained with antibodies against acetylated Tubulin for cilia (magenta), Cep164 for the basal body (green) and Hoechst for nuclei (blue). Cilia occur at different lengths, basal bodies devoid of magenta are unciliated (squared). White lines indicate the leading edge. Scale bars: 10μm. **(B)** Quantification of ciliated cells at various time points in areas away from the leading edge and at the leading edge. 0h: 56.7%±3.3% vs. 31.0%±4.5%, 2h: 58.1%±1.7% vs. 20.2%±4.7%, 4h: 53.3%±2.1% vs. 15.0%±3.0%, 6h: 54.6%±3.1% vs. 7.5%±2.7% (asterisk: p < 0.01). n = 10 fields of view in two independent experiments. Note that the number of cilia at time point 0h at the leading edge is decreased compared to distant cells due to mechanical injury from wounding. **(C)** Analysis of proliferation in cells after wounding. MDCK cells stably expressing Fucci cell-cycle indicators were monitored after 7 days post seeding after scratch wounding. Nuclei of non-proliferating cells in G1 express RFP (red), nuclei of proliferating cells (S/G2/M) express GFP (green). Scale bars: 100μm. **(D)** Quantification of proliferating cells at the leading edge and cells away from the wound over 21 hours after scratch. n = 10 fields of view from two independent experiments.

To investigate the behavior of cilia in MDCK cells during cell migration, we visualized cilia by staining for acetylated Tubulin and the mother centriole (Cep164) immediately after wounding and up to 6 hours later. When we compared cilia at the leading edge, we found that the number of cilia at the leading edge continually decreased over time (Fig 2A and 2B), such that most migrating cells were unciliated after 6h. On the contrary, cilia numbers remained unchanged several cell diameters away from the site of wounding (Fig 2A and 2B). This observation argues against the notion that cilia play a central role in the migratory behavior of epithelial cells during wound closure.

Ift88 has functions in cell-cycle progression that might affect wound closure if cell proliferation contributed to filling the wound after scratching [11]. Therefore, we studied cell proliferation during wound closure, and used retroviral transduction to establish MDCK cells stably expressing the Fucci (fluorescent, ubiquitination-based cell-cycle indicator) transgene [21]. The Fucci reporter is expressed in the nucleus to allow live-cell imaging of cell-cycle progression (S1D Fig). Cells in G1 are characterized by a red fluorescent signal; during S/G2/M-phases a green reporter is expressed. We observed that cells at the leading front of the wound margins remained in G1-phase for more than 12 hours after scratching, long after the time we analyzed migration. By 13h less than 1% of cells were in S/G2/M-phase as shown by green nuclei (Fig 2C and 2D). From these observations we conclude that the disparity in gap closure is not accounted for by differences in cell proliferation.

### Ift88 facilitates cell migration in unciliated MDCK cells

**Fig 3:**
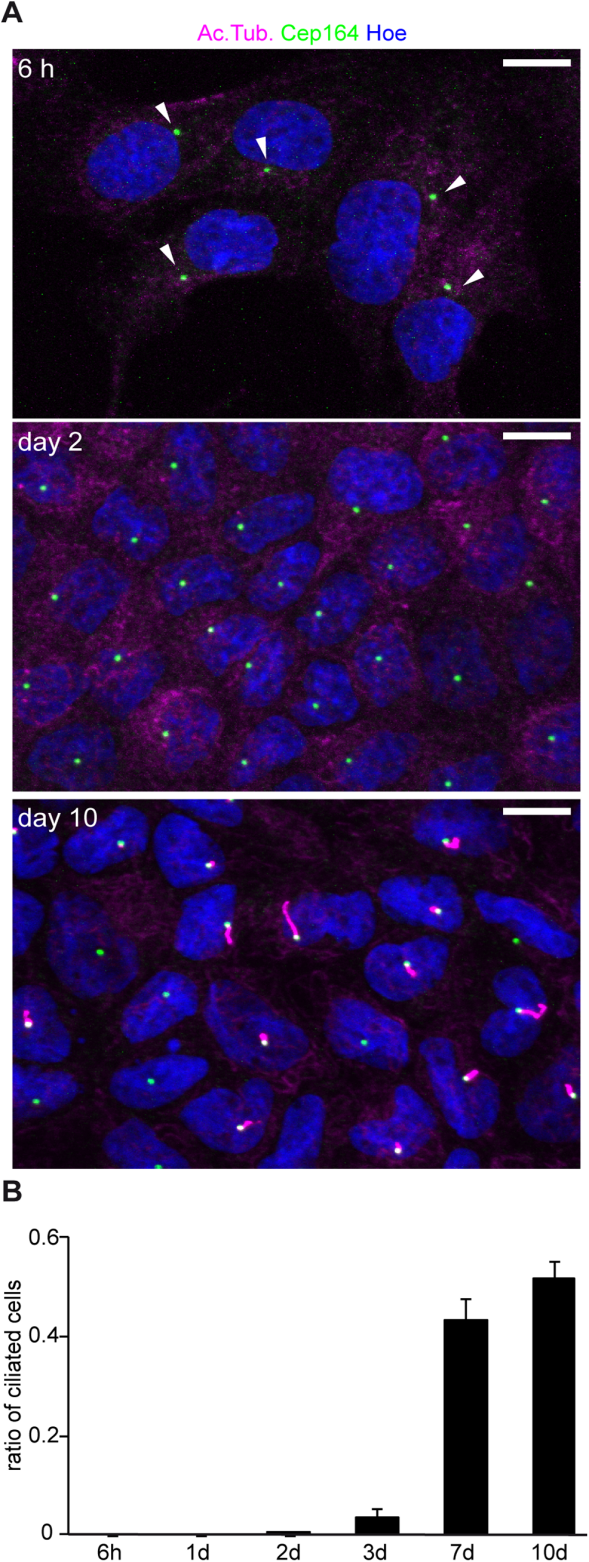
The number of ciliated MDCK cells after seeding increases over time. **(A)** Cells were fixed at respective time points and stained with antibodies against acetylatedTubulin for cilia (magenta), Cep164 for the mother centriole (green) and Hoechst for nuclei (blue). Arrows point to mother centrioles which do not have cilia. **(B)** The number of ciliated cells was quantified over time: 6h: 0.0% ±0.0%, 3420 cells; 1d: 0.0% ±0.0%, 9347 cells; 2d: 0.1% ±0.1%, 18600 cells; 3d: 3.5% ±1.7%, 18309 cells; 7d: 43.2% ±4.7%, 17418 cells and 10d: 51.9% ±3.6%, 17475 cells. n = 3 (32 fields of view per N).

Although cilia are lost over time in cells at the migrating front of the wound margin, a small fraction of cells remained ciliated, therefore making it difficult to exclude that Ift88 affects migration through a non-cell-autonomous function of cilia. Therefore, we took advantage of the fact that MDCK cells form cilia only several days after seeding [22] and examined migration behavior in unciliated cells before this time point. To exactly determine the onset of ciliogenesis, we evaluated the appearance of cilia over time. Basal bodies and cilia were visualized in MDCK cells 6 hours, 1, 2, 3, 7, and 10 days after seeding by staining for Cep164 (basal bodies) and acetylated Tubulin (cilia) (Fig 3A). Visual analysis and automated quantification of ciliogenesis by cytometric imaging revealed that cilia began to appear at day 3 (Fig 3B). Hence, we decided to conduct wound healing experiments on day 2 after seeding, when cilia were present in 0.1% of cells.

**Fig 4:**
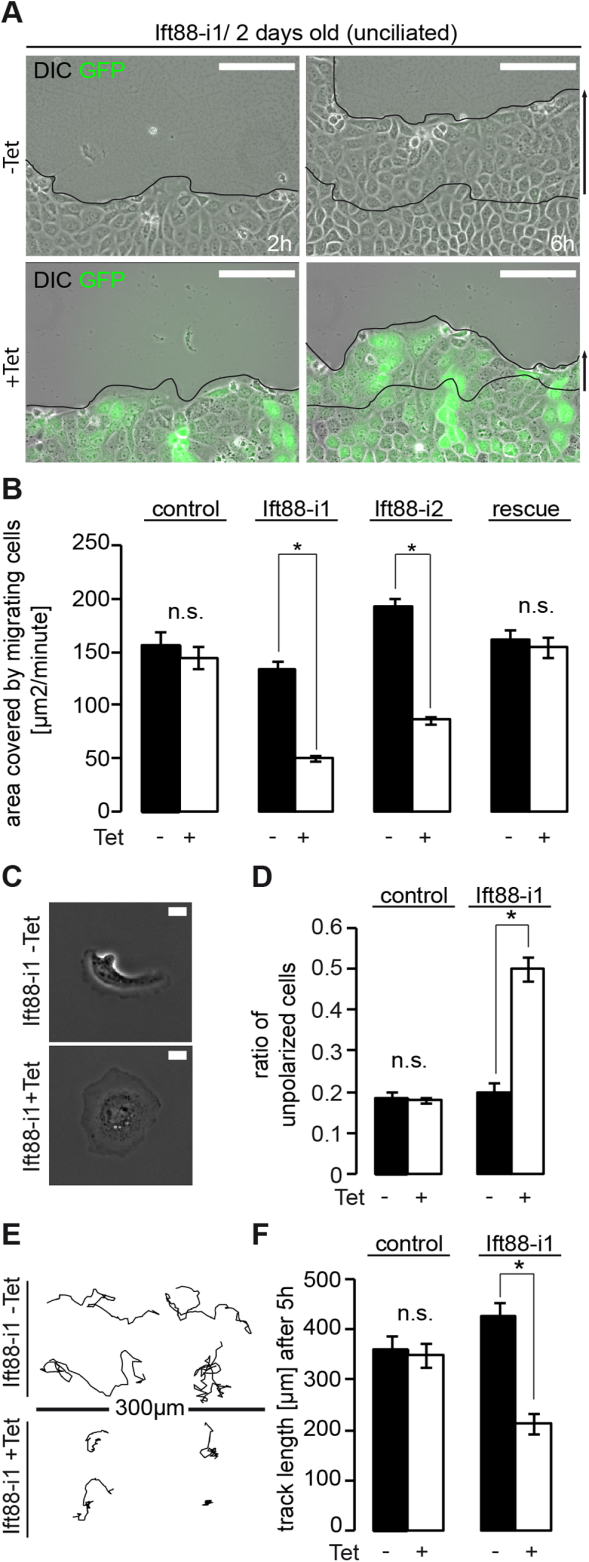
Ift88 expedites cell migration in previously unciliated MDCK cells. **(A)** Ift88-i1 cells were grown to confluency for 2 days (unciliated stage, compare Fig 3) and subjected to wounding. Cells depleted of Ift88 by inducible shRNA (+Tet) migrate more slowly compared with non-induced control cells (-Tet). The leading edge is shown after 2h and 6h. Scale bars: 100μm. **(B)** Quantification reveals no significant reduction of migration speed for a control cell line: -Tet: 157.1 ±12.8 μm^2^/minute vs. +Tet: 144.8 ±10.6 μm^2^/minute (n.s.; p = 0.5). Migration speed is reduced in two independent cell lines after induced depletion of Ift88. Ift88-i1: -Tet: 133.9 ±7.0 μm^2^/minute vs. +Tet: 50.4 ±2.5 μm^2^/minute, p<0.01, Ift88-i2: -Tet: 193.0 ±7.4 μm^2^/minute vs. + Tet: 86.0 ±2.9 μm^2^/minute, p<0.01. Migration speed is restored in Ift88-i1 cells expressing non-degradable Ift88 mRNA (Ift88-i1.rescue): -Tet: 162.0 ±9.8 μm^2^/minute vs. +Tet: 154.6 ±10.0 μm^2^/minute, p = 0.63. All n = 3. **(C)** Sparsely seeded Ift88-i1 cells were stimulated with HGF and the morphology was analyzed. Ift88 depleted cells (+Tet) frequently exhibit an unpolarized pancake shape, while non-induced controls (-Tet) more often show a leading and a trailing edge (A representative cell stained for α-Tubulin and a Golgi marker is shown in S1E Fig). Phase contrast images. Scale bars: 10μm. **(D)** Quantification of the proportion of pancake-shaped cells. No significant increase is seen after exposure to tetracycline in a control cell line: -Tet: 18.3 ±1.9% vs. +Tet: 17.8 ±0.7%, p = 0.87, n = 4, a total of 269 and 392 cells counted. Depletion of Ift88 increases the number of unpolarized cells: -Tet: 19.9 ±2.4% vs. +Tet: 50.2 ±2.9%, p<0.01, n = 4, a total of 454 and 362 cells counted. **(E)** Representative trajectories of single cell migration over 5 hours after stimulation of sparsely seeded cells with HGF (10ng/ml). Scale Bar: 300μm. **(F)** Quantification of track lengths over 5h. In control cells no significant reduction of track lengths is observed after incubation with tetracycline: -Tet: 360.4 ±27.0 μm vs. +Tet: 348.9 ±24.3 μm, p = 0.76, n = 40 tracks from four independent experiments. A reduction is observed in Ift88 depleted cells: -Tet: 426.7 ±28.5 μm vs. +Tet: 213.8 ±21.3 μm, p<0.05, n = 40 tracks from four independent experiments.

While wound closure by migrating cells without cilia was faster compared to ciliated cells (157.1 ±12.8 μm^2^/minute vs. 111.5 ±5.8 μm^2^/minute, p<0.05) we again observed markedly altered migration behavior in both Ift88 depleted cell lines (Ift88-i1 and Ift88-i2,+Tet) compared to control cells (-Tet) (Fig 4A and 4B). Similar to cells that were examined 7 days after seeding, in cells studied after 2 days the migration defect induced by shRNA against Ift88 was reversed by expressing a non-degradable Ift88 rescue construct (Fig 4B). We next asked if the migration defect of Ift88 depleted cells occurs in single migrating cells also, which would suggest a cell autonomous function of ift88. To test this, we analyzed migration in sparsely seeded, unciliated cells. Ift88 deficient cells (+Tet) and non-induced controls (-Tet) were incubated with hepatocyte growth factor (HGF), a chemotactic agent that results in increased migration and the morphological polarization of cells with a broad lamellipodium at the leading edge and a smaller trailing edge [23]. After depletion of Ift88, however, we observed a large number of unpolarized cells, characterized by a “pancake”-shape (Fig 4C and 4D), which has been reported in cells unable to translate directional cues [24]. We then performed timelapse experiments to track individual migration paths (Fig 4E). This revealed a reduction in track length of more than 50% in Ift88 depleted cells (+Tet) (Fig 4F). Taken together, our observations in unciliated epithelial cells demonstrate that Ift88 facilitates effective collective and single cell migration, independently of cilia.

### Ift88 facilitates polarization in migrating cells

**Fig 5:**
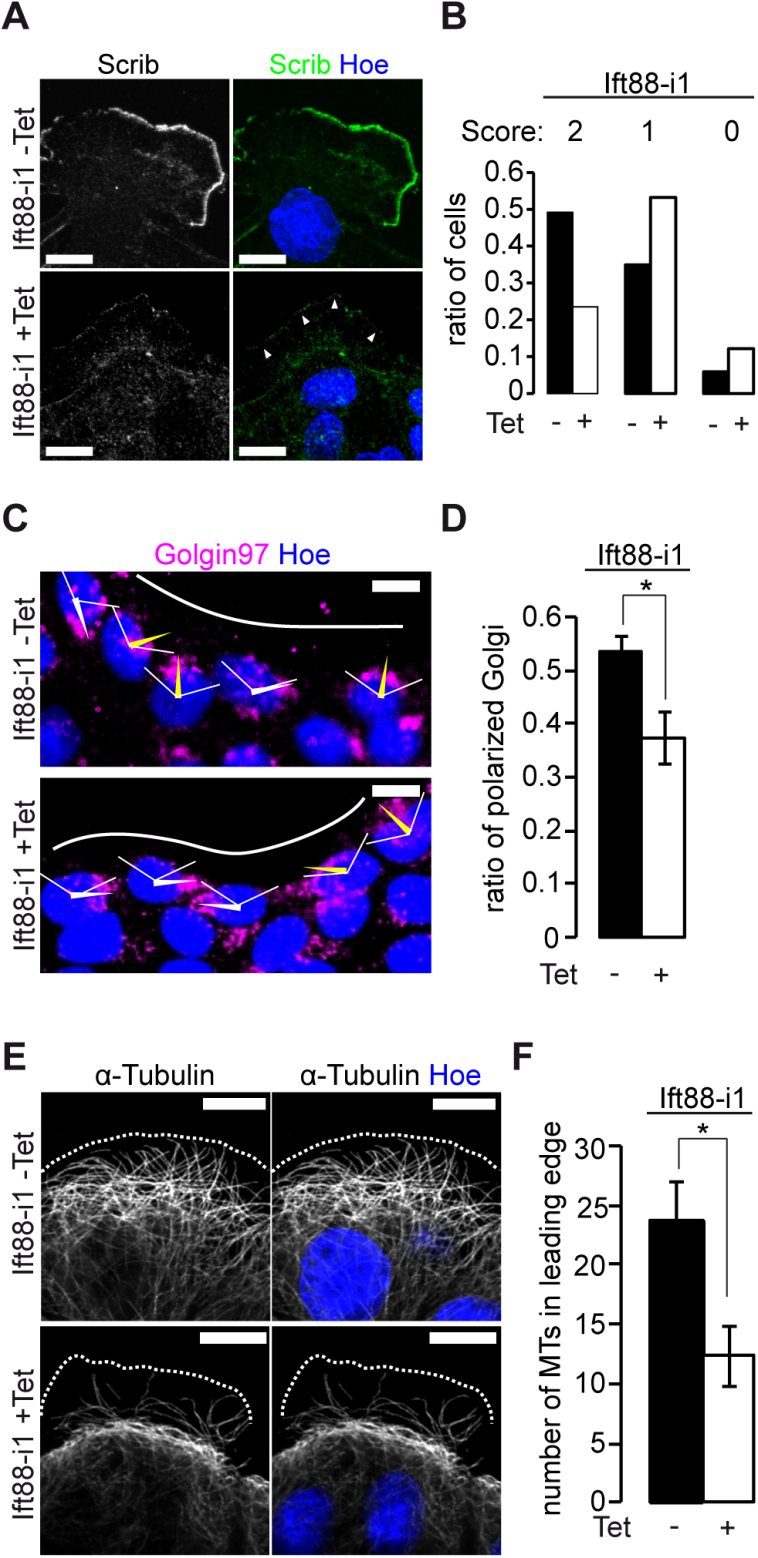
Ift88 facilitates polarization in migrating cells. **(A)** Ift88 depleted (+Tet) or control cells (-Tet) were subjected to wound healing as in Fig 4A. After 6 hours cells were fixed, stained with antibodies against Scrib (green) and Hoechst for nuclei (blue), and the leading edge was imaged. Scale bars: 10μm. White arrows point to the leading edge in absence of Scrib. **(B)** Score based quantification of Scrib localization (0 (not polarized), 1 (partially polarized) or 2 (fully polarized)). After tetracycline induced depletion of Ift88 fewer cells have a fully polarized score (23.5%) compared to the absence of tetracycline (49.2%), n = 3, -Tet 478 cells/ +Tet 468 cells. **(C)** Ift88-i1 cells were subjected to wound healing assay as in Fig 4A. After 6 hours cells were fixed, stained against Golgin-97 for the Golgi apparatus (magenta) and Hoechst for nuclei (blue), and the leading edge was imaged. Red arrows show the direction of polarization of the Golgi apparatus relative to the nucleus and the leading edge within ±60°; white arrows illustrate unpolarized Golgi (not within ±60°). Scale bars: 10μm. White line illustrates the leading edge. **(D)** Quantification of Golgi apparatus polarization (for details see methods) showed a significant decrease of polarized Golgi in Ift88-knockdown conditions (+Tet: 37.5 ±4.8%) compared to non-induced controls (-Tet: 53.6 ±3.3%), p<0.05 (Chi^2^-Test), n = 3, -Tet: 258 cells/ +Tet: 269 cells. **(E)** After 6 hours of migration Ift88-i1 cells were fixed, stained against α-Tubulin (white) and Hoechst for nuclei (blue) showing reduced numbers of MTs in the leading edge in Ift88-knockdown conditions (+Tet). Scale bars: 10μm. **(F)** Quantification of the MTs in the leading edge (conducted in Ift88-i1/ α-Tubulin-YFP cells, see also Fig 6A) revealed significantly fewer MT in Ift88 deficient cells (-Tet: 23.7±3.4 vs. +Tet: 12.4±2.5, p<0.05, n = 10/11 cells in two independent experiments).

The polarization of migrating cells involves the scaffold protein Scribble (Scrib), which localizes at the leading edge and triggers various downstream signaling cascades important for polarized migration [24,25]. Lack of Scrib in epithelial cells has been associated with disordered orientation of the Golgi apparatus in migrating epithelial cells and a defect in cell migration [26,27]. Since we observed a large number of unpolarized cells after depletion of Ift88, we wondered how Scrib localized in these cells. We performed wound healing experiments and stained for Scrib after 6h migration (Fig 5A, for a larger field of view S2A Fig). Applying a scoring system [28], we found fewer cells with complete or partial localization of Scrib at the leading edge in Ift88 depleted cells (+Tet) in comparison with controls (-Tet) (Fig 5B). This difference was not caused by the loss of cell-cell contacts after ift88 depletion since it was observed in confluent cells with comparable amounts of Scrib or actin at the lateral membrane (S2A and S2B Fig). To test the specificity of the Scrib signal we established cells for the tetracycline inducible expression of an shRNA against Scrib. The amount of Scrib as determined by Western blot was strongly decreased after induction by tetracycline (S2C Fig) and the immunofluorescence signal at the lateral membrane was strongly reduced (S2D and S2E Fig). We wondered if the observed lack of Scrib at the leading edge could be explained by an interaction with Ift88, however, we did not detect any Ift88 in lamellipodia of wild type cells (S2F and S2G Fig). Further, we pursued the possibility that Ift88 and Scrib interact at the centrosome and performed co-labelling of γ-Tubulin with Ift88. While we observed co-staining of both proteins at the centrosome in wild type cells, this was also the case in Scrib depleted cells, despite the lack of signal at the lateral membrane. This suggests that the staining of the Scrib antibody at the centrosome is unspecific and argues against a centrosomal interaction between Scrib and Ift88.

In addition, we looked for other evidence of a polarization phenotype in Ift88 depleted cells and examined Golgi polarization. This revealed that significantly fewer of the Ift88 depleted cells had a Golgi orientation within ± 60° perpendicular to the angle of the leading front, with the numbers being close to a random orientation of 33% (Fig 5C and 5D). These observations suggest that Ift88 depleted cells have an impaired capacity to polarize during migration.

### MT dynamics, post translational modification, and MT nucleation are undisturbed in Ift88 depleted cells

We wondered if changes in MT behavior were responsible for the defects in polarization and migration of Ift88 depleted cells and analyzed MT distribution. Interestingly, we observed significantly fewer MTs in the leading edge of Ift88 depleted compared to non-induced control cells (Fig 5E and 5F). We wondered if MT stability was affected and assessed the amount of posttranslational modification in migrating Ift88 deficient cells [29]. This revealed no difference in the amount of acetylated Tubulin (data not shown) compared to control cells.

**Fig 6:**
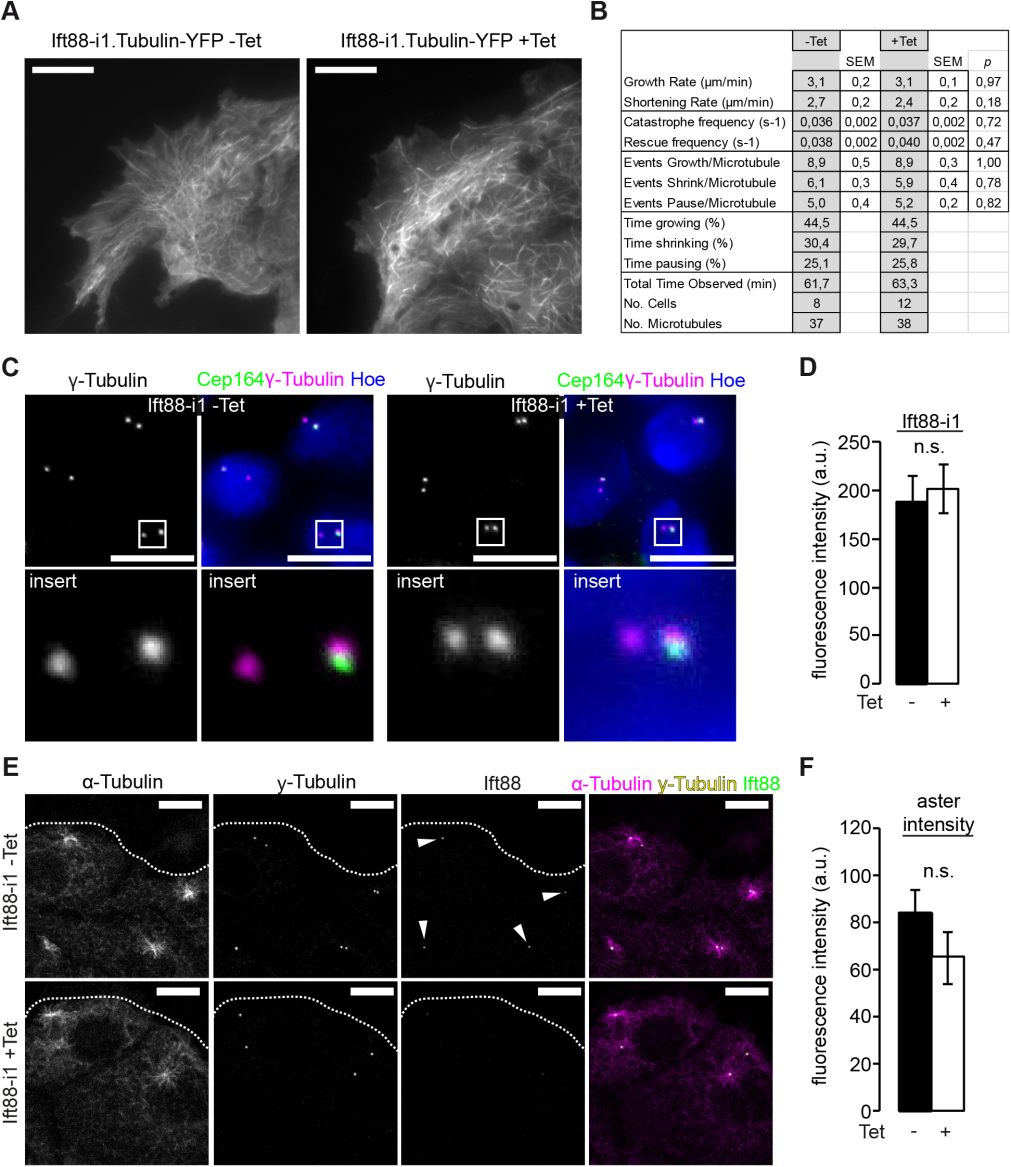
Ift88 does not influence MTOC activity. **(A)** Migrating Ift88-i1 cells with stably transduced α-Tubulin-YFP (Ift88-i1.Tubulin-YFP) were subjected to TIRF microscopy and MT were assessed. The number of MT probing into the leading edge is reduced upon Ift88-knockdown compared to non-induced controls, but **(B)** no changes in MT dynamic instability were observed. **(C)** The amount of γ-Tubulin at the centrioles appears similar in Ift88-knockdown cells (+Tet) compared to non-induced controls (-Tet). Lower panel shows magnifications of the white squares. **(D)** Quantification of γ-Tubulin fluorescence intensity reveals no significant difference between -Tet (188.3 ±27.3 a.u.) and +Tet (201.5 ±25.1 a.u.) conditions. p = 0.72, n = 6, 19 fields of view each, 556/461 cells. **(E, F)** Migrating Ift88-i1 cells as in Fig 4A were subjected to MT depolymerization with nocodazole and subsequently MT regrowth was analyzed. Dotted line illustrates the leading edge. No significant difference was observed in aster intensity (-Tet: 84.0 ±9.9 a.u. vs. +Tet: 65.2 ±11.0 a.u., p = 0.27, n = 3, total of 33/34 centrosomes). Ift88-knockdown was confirmed by staining for Ift88 comparing fluorescence intensity at the mother centriole in ±Tet conditions (-Tet: 84.1 ±11.8 a.u vs. +Tet: 34.8 ±11.0 a.u., p<0.05, n = 3). Scale bars: 10μm.

MTs undergo dynamic interactions between their plus-ends and the cellular cortex [30]. This process is referred to as dynamic instability and is characterized by elongation of MT tips, shortening and pausing. In the cilium, Ift88 is transported along MTs as part of the multi-protein Ift complex B towards the plus-end by kinesin-2. Loss of the kinesin-2 subunit Kif3a is associated with altered MT dynamics [19], so we wondered if similar alterations occur in Ift88 depleted cells. We transduced our inducible Ift88-i cells to stably express α-Tubulin-YFP and measured MT dynamic instability in migrating Ift88 depleted cells (+Tet) by total internal reflection microscopy (Fig 6A and 6B, S1 Video). We observed no differences in any of the measured parameters of MT dynamic instability compared to control cells (-Tet) (Fig 6B), suggesting a divergent function of Ift88 and Kif3a in MT tip behavior. Indeed, in immunostainings of migrating wild type cells, we always found Ift88 strongly concentrated at the mother centriole but never at the leading edge (S2F and S2G Fig) which differs from previous observations with Kif3a [19].

A main determinant of MT geometry apart from dynamic instability and posttranslational modification is MT nucleation, the majority of which occurs at the centrosome, also referred to as the microtubule organizing center (MTOC) [31]. This requires γ-Tubulin which assembles into the γ-Tubulin ring complex (γ-TuRC) to nucleate MT polymerization. In Ift88 deficient mitotic cells, γ-Tubulin fails to localize at the centrosome, presumably because of disturbed minus-end directed transport of the dynein-Ift88 complex, and is associated with the lack of astral MTs [10]. We quantified γ-Tubulin levels at the mother centriole during cell migration, but found no difference in the γ-Tubulin fluorescent signal between Ift88 depleted (+Tet) and control cells in interphase (-Tet) (Fig 6C and 6D). We then analyzed de novo MT nucleation at the MTOC, after depolymerization of MTs with nocodazole (Fig 6E). Morphologically no difference was found in the shape or size of the MT asters growing at the MTOCs between Ift88 depleted (+Tet) and control cells (-Tet) (Fig 6E). Of note, no focal extra-centrosomal sites of MT nucleation were observed. Quantification of MT signals from newly forming asters (Fig 6F) revealed no significant difference between Ift88 depleted (+Tet) and control cells (-Tet). Taken together, Ift88 depletion is associated with decreased numbers of MT at the leading edge of migrating cells. Yet, we found no difference in MT dynamics, post-translational modification of MTs or MT nucleation, suggesting that other factors account for this disparity.

## Discussion

We show a cilia independent function of Ift88 in epithelial cell migration. Cell migration is a central process in biology [18]. In adherent single cell organisms it is vital for movement along a surface or substrate. In vertebrates cell migration is an integral part of embryonic development and plays a role in tissue homeostasis, as exemplified by migrating enterocytes that travel from crypts towards the tips of villi [32]. In pathological conditions migration is crucial for injury response, for instance during wound healing, or cancer, where mutant cells migrate out of their natural environment [33]. Cell migration is a coordinated process that involves highly dynamic changes of MTs and the actin cytoskeleton to enable propulsion. In addition it requires the sensing of directional cues to ensure the spatial orientation of migrating cells and thus directionality [18].

Cell migration has been linked to cilia in numerous cell types: In fibroblasts cilia orient towards the migrating front during wound repair after scratching [16]. In the developing nephron of the zebrafish interfering with Ift88 by antisense morpholinos impairs collective cell migration [34]. During brain development cilia localize to the anterior part of migrating neuronal cells [35], and corneal epithelial cells lose their cilia after the completion of eye development, yet cilia reemerge during wound repair after injury [36]. Our findings in renal derived MDCK cells demonstrate a different behavior: at the resting state confluent cells display an apical cilium. However, after establishing a scratch wound in the ciliated epithelial layer we find that most cilia in the cells at the migrating front get absorbed within a few hours. Our findings are in agreement with observations after acute renal injury where cilia on renal tubular cells are lost or shortened early after the insult [37,38], but differ from observations in zebrafish, where cilia are maintained at the leading edge of a repairing wound in the pronephros for 4–6h [39]. Overall, these diverging findings suggest that the role of cilia in injury repair is context specific rather than universal.

In the current study we find that highly effective depletion of Ift88 impairs migration of cells that are analyzed prior to the onset of ciliogenesis. We have previously described that depletion of the kinesin-2 subunit Kif3a, which is involved in the transport of the Ift B complex, including Ift88, along the ciliary shaft, similarly impairs the migration of unciliated cells [19]. Because of the functional interaction between Kif3a and Ift88 in cilia we would have expected the underlying mechanisms to be analogous, but found this not to be the case. While in both, Ift88 and Kif3a depleted cells, migration was similarly impaired, appearance and behavior of MTs were different. In Kif3a deficient cells MTs at the leading edge grow in oblique angles into a thinned lamellipodium. In Ift88 depleted cells, however, directional MT growth into the lamelipodium was normal, only the numbers of MTs were fewer. A striking difference was observed in the dynamic behavior of MT tips. MT tip movements were unchanged in IfT88 deficient cells, but appear frozen after Kif3a depletion [19]. While Ift88 and Kif3a functionally interact in cilia, our observations suggest differing functions for Ift88 and Kif3a outside the cilium. This conclusion is supported by our observation that Kif3a but not Ift88 is detected in lamellipodia of migrating cells.

In light of the normal MT dynamics what could be the reason for the reduced number of MT at the lamellipodium in Ift88 depleted cells? MTs are mostly nucleated at the centrosome [17]. This requires the recruitment of the γ-Tubulin ring complex which serves as a template for new MT to form. During mitosis γ-Tubulin accumulates at the centrosome in an Ift88 dependent process. The lack of Ift88 leads to a failure to assemble astral MTs [10]. We didn't find differences in γ-Tubulin levels at the centrosomes of Ift88 depleted interphase cells suggesting different roles of Ift88 in mitosis and interphase. Similarly, MT nucleation as observed after nocodazole treatment was normal. MT nucleation also occurs at extra-centrosomal sites such as the Golgi apparatus. However, we did not see any differences of extra-centrosomal MT nucleation in our nocoadazole assays to explain the reduced number of MTs in the lamellipodium. We explored the possibility that posttranslational modifications of MT are altered in Ift88 depleted cells. Yet, in contrast to cells completely lacking Ift88 [29], we did not observe increased amounts of acetylated Tubulin. This may indicate that depletion of Ift88 –as opposed to a complete lack of it–does not affect MT stability and consequently cannot explain the migration defect.

Migration deficient Ift88 depleted cells were characterized by the inability to polarize. Single cells frequently were pancake shaped; the orientation of the Golgi apparatus was randomized in cells at the migrating front and Scrib localization at the lamellipodium was disturbed. Scrib is part of a cell surface complex including the proteins Discs Large (Dlg) and Lethal Giant Larvae (Lgl). In polarized epithelia the Scrib complex is a determinant of the basolateral domain [40]. In migrating cells Scrib induces the activation of Rac-GTPases, aPKC and GSK3 to effect the polarization of MT [18]. The mechanisms of Scrib recruitment to the leading edge of migrating cells have not been clarified but our data show that Ift88 is required for the localization of Scrib. On the other hand the lack of Scrib at the leading edge may explain the reduced number of MT in the lamellipodia of Ift88 depleted cells.

Ift88 has a number of extraciliary functions including cell-cycle regulation, mitosis, MT stability, and MT orientation. However, a mechanistic understanding bringing these functions together is lacking. A hint at what this basic function might be comes from theoretical considerations and from studies in lymphocytes [12,41]. Ift20 is a member of the Ift B complex and is required for cilia formation. In lymphocytes Ift20 plays a role in a recycling process that targets the T-cell receptor to the immunological synapse [12]. It has been postulated on structural grounds that Ift proteins resemble vesicle coat proteins [41]. Vesicular transport is important for cell migration: Golgi derived vesicles and recycling endosomes are transported along MT to the lamellipodium, where they release membrane particles and proteins to enable propulsion [18,42]. It will be interesting to investigate in the future if vesicular transport is mediated by Ift88 and its associated Ift proteins and how that could contribute to cell migration and possibly the cell-cycle and mitosis.

## Methods and Materials

### Cell culture and transgenic cell lines

MDCK II cells were kindly provided by Kai Simons (Dresden, Germany) [43]. MDCK cells were grown at 37°C, 21% O_2_ and 5% CO_2_, and maintained in Dulbecco’s modified Eagle’s medium (DMEM), 10% FBS, and 1% penicillin/streptomycin. For cells containing fusion proteins (pLXSN vector) 2.5 mg/ml geneticin was added. A lentiviral system was used for the inducible Ift88-knockdown [20]. MDCK cells were first transduced with lentivirus encoding the tetracycline sensitive tTR-KRAB repressor (pLVTH vector). A second transduction step followed with lentivirus encoding a shRNA against Ift88 plus a GFP reporter (pLVTH vector), both under the control of tTR-KRAB [44]. Control cells were obtained in a similar process, except that lentivirus for the second transduction was prepared with an unspecific shRNA against luciferase or empty vector [19];Target sequences for Ift88-i1 (5ʹ-GAAGGCAGCTGAATTCTAT-3ʹ) and Ift88-i2 (5ʹ-GAGCTAGCAAATGATCTGG-3ʹ) [45]. The shRNA against Scrib has been described elsewhere [46]. To induce knockdown conditions ± 5μg/ml tetracycline was added after seeding of cells and renewed with medium change every two days. For the single cell migration assays, cells were incubated ± 5μg/ml tetracycline 2 days prior seeding for the experiment.

For the Ift88-1.rescue cell line, the GFP reporter cassette of the pLVTH vector containing shRNA “Ift88-i1” was replaced by hIft88. Knockdown resistant hIft88 was generated through site-directed mutagenesis primer: (5'-ATTATGAGAAAGCCGGTGAGTTCTATAAAGAGGCCCTGAG-3ʹ) and lentivirally transduced in Ift88-i1 cells. The α-Tubulin-YFP transduction has been described [19]. Fucci-vectors (Fucci-G1-Orange-mKO2-hCdt1, Fucci-S/G2/M-Green-mAG1-hGem) were obtained from Clontech Laboratories, Inc (Mountain View, USA). PCR cloning into pLXSN was performed with fw-primer (5ʹ-CGCGGGGAATTCGCCACC ATGGTGAGTGTGATTAAA-3ʹ (Fucci-G1), 5ʹ-CGCGGGGAATTCGCCACC ATGGTGAGCGTGATCAAG-3ʹ (Fucci-G2/S/M)) including an EcoRI restriction site and rw-primer (5ʹ-CGCGGG GGATCCTCATTAGATGGTGTCCTGGTC-3ʹ (Fucci-G1), 5ʹ-CGCGGGGGATCCTCATTACAGCGCCTTTCTCCG-3ʹ (Fucci-G2/S/M)) with a BamHI restriction site, and then cloned into pLXSN. Both vectors were retrovirally transduced in MDCK cells resulting in most cells carrying FucciG1 and FucciS/G2/M.

### Wound healing and migration of subconfluent cells

For wound healing assays, MDCK cells were plated and grown to confluency on Ibidi μ-dishes (Ibidi, GmbH, Munich, Germany) coated with collagen A (Biochrom AG, Berlin, Germany). For quantification of migration speed, cells were incubated for 2 (unciliated) or 7–8 days (ciliated) ± 5μg/ml tetracycline and wounded using a micropipette. Cells were imaged using a Nikon Biostation IM (Nikon, Melville, USA), which includes a CO_2_ incubation chamber equipped with heating unit and a motorized xy-table. Migration speed was calculated from 4 hours of cell migration between 1 and 8 hours after wounding. The semi automatic tracking properties of NIS ElementsSystem (Nikon, Melville, USA) software were used to quantify 5–6 fields of view per n. Migration assays with platings of approximately sparse 5000 cells were conducted with the same setup. Cells were treated with 10 ng/ml HGF (Hepatocyte Growth Factor, ImmunoTools, Friesoythe, Germany). Tracking of representative cells were made with Imaris software (version 7.1, Bitplane, Zurich, Switzerland).

### Imaging

Confocal microscopy was performed using a LSM 5 Life Duo equipped with C-Apochromat 63×/1.3 NA (water) and 100x/1.46 NA (oil) objectives (all Carl Zeiss MicroImaging GmbH, Jena, Germany). Excitation of the fluorophores (Hoechst 33342, Alexa-488, YFP, Cy3, Cy5) was performed at 405, 488, 512, 561 and 633 nm respectively. For detection of the emission signal at specified ranges, the spectral meta detector or normal photomultiplier channels were used with BP filter 420–480, BP 505–530, LP 530, BP 575–615, LP 650 nm. Image analysis and imaging of Ift88-i1/α-Tubulin–YFP cells for MT dynamics measurements were performed with a Laser-TIRF 3 system in temperature controlled conditions on an inverted TIRF-microscope (Axiovert 200 Microscope with objective Apochromat 100x/1.46 oil, Carl Zeiss MicroImaging, Jena, Germany). MDCK.Ift88-i1/α-Tubulin-YFP cells were plated into μ-dishes with a glass bottom (Ibidi GmbH, Munich, Germany) incubated for two days in ± tetracycline conditions, scratched, and mounted on an incubation stage (TokaiHit, Shizuoka-ken, Japan) with 5% CO_2_ incubation and stable temperature at 37°C. Representative leading edge cells of each condition were imaged 6 hours after wounding at 5s intervals using the 514nm laser. MT dynamic instability parameters were calculated with Excel (Microsoft Corporation, Redmond, USA).

### Immunofluorescence

2D cultures stainings were fixed using 4% paraformaldehyde or methanol/acetone (1:1) depending on the antibody. Cells were permeabilized with 0.1% Triton X-100 in PBS and incubated in blocking solution (5% horse serum or 0.2% gold fish gelantine). Cells were subsequently incubated with 1:1000 monoclonal mouse anti-acetylated-Tubulin antibody (Sigma-Aldrich, Saint Louis, USA), 1:200 monoclonal mouse anti-α-Tubulin (Sigma-Aldrich, Saint Louis, USA), 1:200 mouse anti-γ-Tubulin (Sigma-Aldrich, Saint Louis, USA), 1:200 rabbit-anti-Ift88 (ProteinTech Group Inc, Chicago, USA), 1:100 goat anti-Scrib (C20, SantaCruz, Dallas, USA), 1:200 monoclonal mouse anti-Golgin-97 (Molecular Probes Europe, Leiden, The Netherlands), 1:200 rabbit anti-GM130 (Sigma-Aldrich, Saint Louis, USA), anti rabbit-Cep164 (gift from Erich A. Nigg) and Hoechst 33342 (Life Technologies GmbH, Darmstadt, Germany). Antibodies were visualized using Cy3-, Cy5- or Alexa-488-labelled secondary antibodies at a dilution of 1:1000 (Jackson Immunoresearch, West Grove, USA).

### Cilia quantification (Imaging Cytometry)

In Fig 2 cilia quantification was achieved by manually counting the number of ciliated cells. Cilia quantification in Fig 3 was completely automated. Determination of ciliated cells was conducted by analysis of immunofluorescence stainings using an Olympus Scan^R screening station (high content screening). For each time point and experiment the microscope scanned automatically two independent square areas, consisting of 16 neighbouring field of views (40x magnification). After image acquisition, data has been analysed with the Scan^R analysis software using a customized ciliogenesis-assay [47].

### Golgi polarization, scoring of Scrib at the leading edge and microtubule quantification

Cells were plated and grown to confluency. After two days, cells were scratched, then incubated for 6 hours of migration, and finally fixed and stained as described above. For quantification of Golgi polarization 10 fields of view from two independent experiments were randomly selected from areas of wound healing, imaged, and analyzed with Image J software (version 1.39u). “Polarized” Golgi were considered those within within ±60° in the direction of cell migration. Scrib localization at the leading edge was examined in two independent experiments as reported by Shin et. al. [28]. Briefly, twenty randomly fields were scored for -Tet and +Tet conditions: 0 points for missing localization of Scrib at the leading edge, 1 point for partial localization, 2 points for complete localization. MTs were quantified using the Ift88.i15/α-Tubulin-YFP cell line counting the number of MTs per cell.

### Nocodazole experiments

MDCK.Ift88.i1 cells were grown on glass cover slips for two days with or without tetracycline, scratched, and after 6 hours of cell migration subjected to nocodazole treatment. Nocodazole (10μg/ml) was added to the medium for 2 hours, cells were then incubated for 5 minutes after nocodazole wash out, and subjected to the methanol/acetone staining protocol as described above. Cells were stained against γTubulin, α-Tubulin and Ift88. Imaging of z-stacks (17–20 images, 90x90μm) was conducted with the LSM confocal microscope. Maximum intensity projections were created with Zen 2012 (Carl Zeiss MicroImaging GmbH, Jena, Germany) software using the “Histo”-application to measure the aster (α-Tubulin) intensity in a circle (3 μm^2^) around the mother centriole. Only leading edge cells were measured.

### Western Blot

The following antibodies were used: anti-Flag (Sigma-Aldrich, M2, Saint Louis, USA), anti-Actin (Sigma-Aldrich, A1978, Saint Louis, USA), anti-acetylated-Tubulin (Sigma-Aldrich, T6793, Saint Louis, USA), anti γ-Tubulin (Sigma-Aldrich, Saint Louis, USA), anti-Ift88 (gift from Bradley Yoder) and anti-Ift88 (ProteinTech Group, No.:13967–1 AP, Chicago, USA). Cells were grown in 10 cm cell culture dishes.

### Data analysis and statistics

Statistical analysis was carried out in EXCEL (Microsoft Corporation, Redmond, USA). Statistical significance was calculated by unpaired t-test when not further indicated or Mann-Whitney Rank Sum test. All values are given as mean ± s.e.m. (standard error of the mean). P values of less than 0.05 were considered to be statistically significant.

## Supporting Information

S1 Fig **(Figs A and B)** Western Blot analysis of Ift88-i1 and Ift88-i2 cell lines after incubation with or without tetracycline for five days. Ift88 is strongly depleted in +Tet conditions. Actin demonstrates equal loading. **(Fig C)** Western Blot analysis of the Ift88-i1.rescue cell line. Incubation with tetracycline for two days, shows expression of shRNA resistant flag-tagged Ift88. Actin demonstrates equal loading. **(Fig D)** Live-cell time-course experiment with MDCK.FucciG1 (red).FucciS/G2/M (green) cells to verify expression of both Fucci-constructs. Scale bars: 100μm. **(Fig E)** HGF stimulated sparsely seeded MDCK cells stained against α-Tubulin (magenta) the Golgi (green) and the nucleus (blue). The cell shows a leading and a trailing edge. Scale bar: 10μm.
(TIF)

S2 Fig **(Fig A)** Migrating Ift88-i cells were stained against Scrib (white) and Hoechst for nuclei (blue). Scrib localizes to the leading edge of -Tet cells while this signal is reduced in +Tet conditions. Scale bars: 10μm. **(Fig B)** The same cells were stained against Phalloidin (magenta) for actin, α-Tubulin (green) and Hoechst for nuclei (blue). Scale bars: 10μm. **(Fig C)** Western Blot analysis of the Scrib-i cell line. Incubation with tetracycline for two days shows Scrib knockdown in +Tet conditions. β-Catenin demonstrates equal loading. **(Fig D)** Scrib-i cells were stained against γ-Tubulin (magenta) for centrosomes and Scrib (green). Scale bars: 10μm. **(Fig E)** Quantification of Scrib signal at the centrosome (-Tet: 81.7 ±5.5 a.u. vs. +Tet: 83.2 ±14.2 a.u., p = 0.93, n = 4 fields of view in two independent experiments, total of 74/83 centrosomes). **(Fig F)** Migrating MDCK cells were stained against Ift88 (magenta), γ-Tubulin for the centrosome (green) and Hoechst for nuclei (blue). Ift88 localizes to one of the two centrioles. Dotted lines correspond to the leading edge. Scale bars: 10μm. The insert shows a magnification of the white square in the merged image. **(Fig G)** Maximum intensity projection of a confocal z-stack (34 planes, plane distance 0.2 μm, pinhole set to 1μm) hsows Ift88 (green) signal al the centriole, but no signal at the leading edge associated with microtubules (magenta). Scale bars: 10μm.
(TIF)

S1 Video MT dynamics in migrating MDCK.Ift88-i1/α-Tubulin-YFP cells. MDCK.Ift88-i1 cells expressing α-Tubulin-YFP (Venus) were incubated with or without tetracyline and MTs were observed in migrating cells six hours after wounding to quantify MT dynamics. No difference in MT dynamics can be seen.
(MPG)

## Abbreviations

Ift: intraflagellar transport
