# Supplementary figures and images for: A Cilia Independent Role of Ift88/Polaris during Cell Migration
Source: PLoS One. 2015 Oct 14;10(10):e0140378. doi: 10.1371/journal.pone.0140378 (PMC4605505; doi:10.1371/journal.pone.0140378)

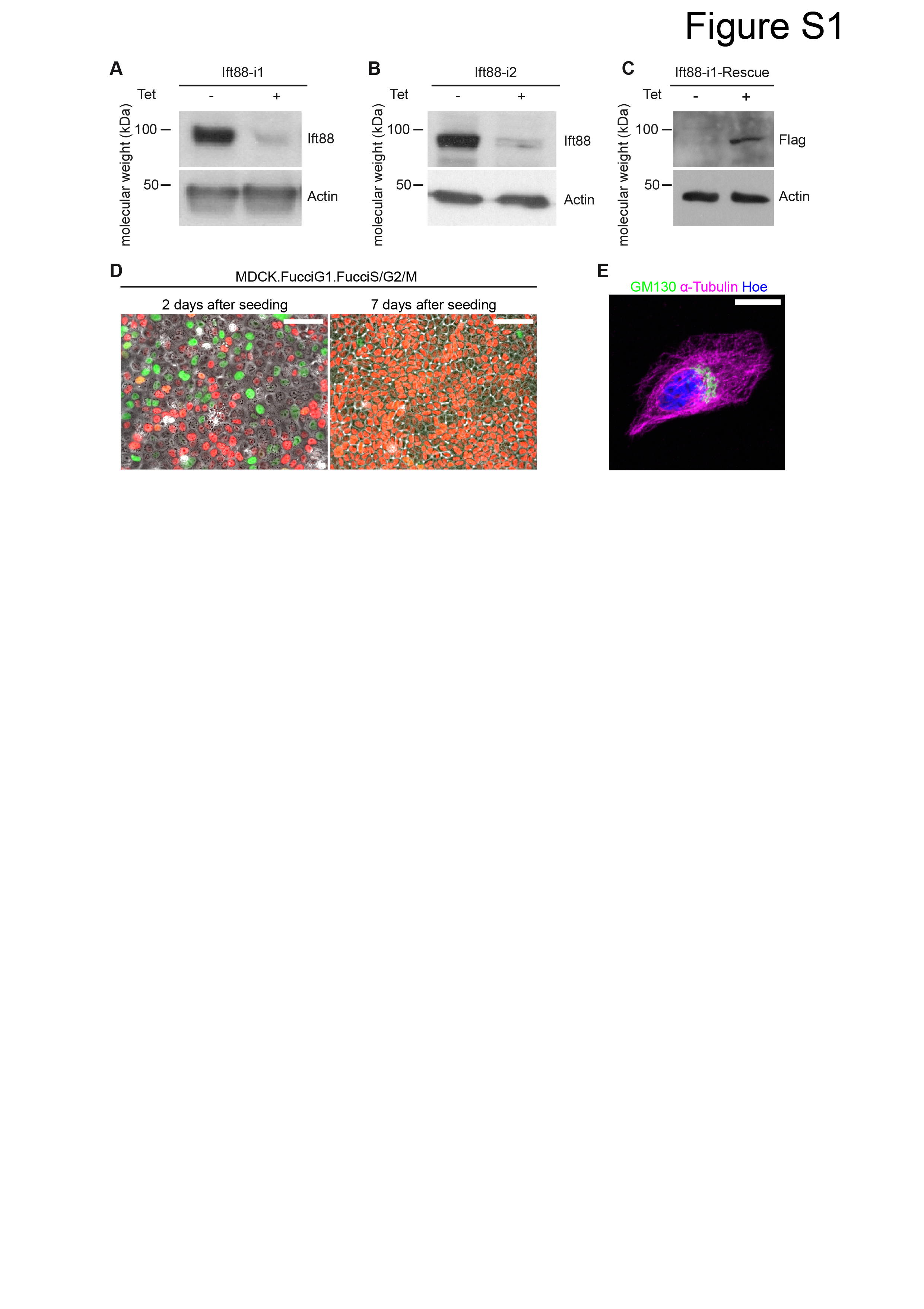

Supplement: S1 Fig — (Figs A and B) Western Blot analysis of Ift88-i1 and Ift88-i2 cell lines after incubation with or without tetracycline for five days. Ift88 is strongly depleted in +Tet conditions. Actin demonstrates equal loading. (Fig C) Western Blot analysis of the Ift88-i1.rescue cell line. Incubation with tetracycline for two days, shows expression of shRNA resistant flag-tagged Ift88. Actin demonstrates equal loading. (Fig D) Live-cell time-course experiment with MDCK.FucciG1 (red).FucciS/G2/M (green) cells to verify expression of both Fucci-constructs. Scale bars: 100μm. (Fig E) HGF stimulated sparsely seeded MDCK cells stained against α-Tubulin (magenta) the Golgi (green) and the nucleus (blue). The cell shows a leading and a trailing edge. Scale bar: 10μm. (TIF) [file pone.0140378.s001.tif]

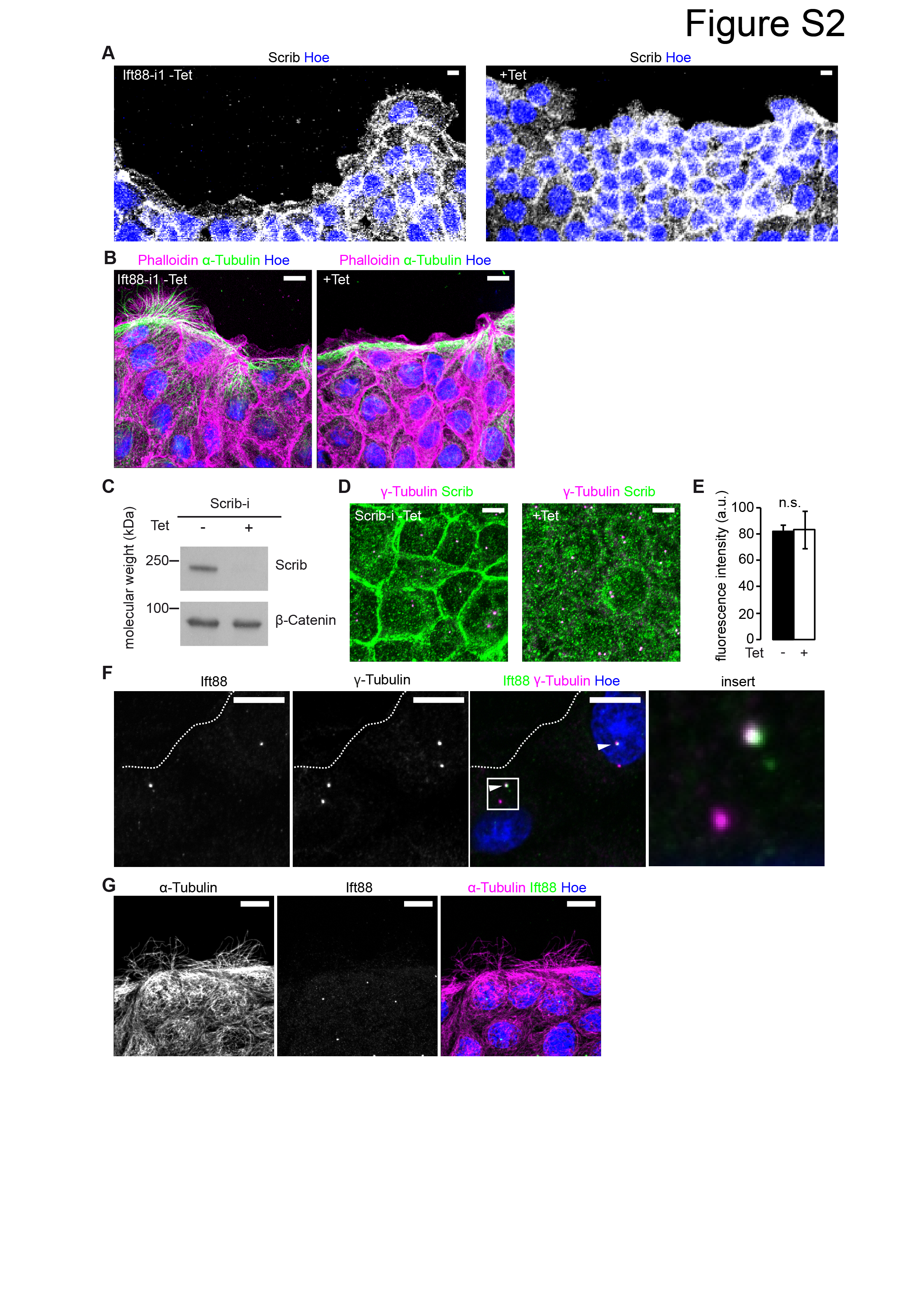

Supplement: S2 Fig — (Fig A) Migrating Ift88-i cells were stained against Scrib (white) and Hoechst for nuclei (blue). Scrib localizes to the leading edge of -Tet cells while this signal is reduced in +Tet conditions. Scale bars: 10μm. (Fig B) The same cells were stained against Phalloidin (magenta) for actin, α-Tubulin (green) and Hoechst for nuclei (blue). Scale bars: 10μm. (Fig C) Western Blot analysis of the Scrib-i cell line. Incubation with tetracycline for two days shows Scrib knockdown in +Tet conditions. β-Catenin demonstrates equal loading. (Fig D) Scrib-i cells were stained against γ-Tubulin (magenta) for centrosomes and Scrib (green). Scale bars: 10μm. (Fig E) Quantification of Scrib signal at the centrosome (-Tet: 81.7 ±5.5 a.u. vs. +Tet: 83.2 ±14.2 a.u., p = 0.93, n = 4 fields of view in two independent experiments, total of 74/83 centrosomes). (Fig F) Migrating MDCK cells were stained against Ift88 (magenta), γ-Tubulin for the centrosome (green) and Hoechst for nuclei (blue). Ift88 localizes to one of the two centrioles. Dotted lines correspond to the leading edge. Scale bars: 10μm. The insert shows a magnification of the white square in the merged image. (Fig G) Maximum intensity projection of a confocal z-stack (34 planes, plane distance 0.2 μm, pinhole set to 1μm) hsows Ift88 (green) signal al the centriole, but no signal at the leading edge associated with microtubules (magenta). Scale bars: 10μm. (TIF) [file pone.0140378.s002.tif]
